# A Survey of the Use of Modeling, Simulation, Visualization, and Mapping in Public Health Emergency Operations Centers during the COVID-19 Pandemic

**DOI:** 10.3390/ijerph21030295

**Published:** 2024-03-02

**Authors:** Ali Asgary, Mahbod Aarabi, Shelly Dixit, He Wen, Mariah Ahmed, Jianhong Wu

**Affiliations:** 1 Disaster & Emergency Management, School of Administrative Studies & York Emergency Mitigation, Engagement, Response, and Governance Institute & ADERSIM, York University, Toronto, ON M3J 1P3, Canada; aarabi@yorku.ca (M.A.); dixits@my.yorku.ca (S.D.); mariah.ahmed81@gmail.com (M.A.); 2 Faculty of Engineering—Civil and Environmental Engineering Department, University of Alberta, Edmonton, AB T6G 2E2, Canada; hwen7@ualberta.ca; 3 York Emergency Mitigation, Engagement, Response, and Governance Institute & Laboratory for Industrial and Applied Mathematics, York University, Toronto, ON M3J 1P3, Canada; wujh@yorku.ca

**Keywords:** survey, digitalization, emergency operations center, EOC, PHEOC, COVID-19, emergency management, pandemic management, modeling, simulation, visualization, mapping

## Abstract

The COVID-19 pandemic has significantly changed life and work patterns and reshaped the healthcare industry and public health strategies. It posed considerable challenges to public health emergency operations centers (PHEOCs). In this period, digital technologies such as modeling, simulation, visualization, and mapping (MSVM) emerged as vital tools in these centers. Despite their perceived importance, the potential and adaptation of digital tools in PHEOCs remain underexplored. This study investigated the application of MSVM in the PHEOCs during the pandemic in Canada using a questionnaire survey. The results show that digital tools, particularly visualization and mapping, are frequently used in PHEOCs. However, critical gaps, including data management issues, technical and capacity issues, and limitations in the policy-making sphere, still hinder the effective use of these tools. Key areas identified in this study for future investigation include collaboration, interoperability, and various supports for information sharing and capacity building.

## 1. Introduction

Triggered by the COVID-19 pandemic, Public Health Emergency Operations Centers (PHEOCs) were activated around the globe, putting their functionality and capabilities to the test [1]. As a focal point of emergency management, PHEOCs handle coordination, communication, management, and information gathering and analysis in complex and evolving situations. They also monitor the availability of health resources, the cost-effectiveness of public health strategies, public opinion, and ethical concerns [2]. Many PHEOCs apply digital tools [3] such as modeling, simulation, visualization, and mapping (MSVM) that are crucial for their operational areas and decision-making processes [4,5]. PHEOCs also benefit from the use of MSVM in organizational management operations, emergency response, academic research, and public communication during the pandemic [6,7,8].

Despite these advancements, PHEOCs encounter several challenges in applying MSVM tools. Notably, many PHEOCs had not scaled up their Incident Command System (ICS) or Incident Management System (IMS) to the magnitude of the COVID-19 pandemic [9], leading to gaps in training and preparedness. In addition, rapid shifting to virtual operations underscored the critical need for digital and information technology supports and continuity of operations [10]. The pandemic response was further complicated by the transition to remote work and the reliance on Virtual Emergency Operations Centers (VEOCs), which highlighted the need for more accessible MSVM. A final challenge to note was the lack of “opportunistic information”, which could have helped address barriers to maintaining mutual awareness with respect to information and needed tools, uneven workload, and disrupted communication. These gaps are now being recognized, and demand for more accessible modeling and visualization tools in public health is increasing [11,12].

MSVM tools combine various technologies to enhance understanding and analyze and visualize data, leading to more effective and quicker decision-making. These tools encompass mathematical models, simulation software, visualization tools, and mapping platforms. Mathematical models are a representation of objects and their behaviors using mathematical expressions, facilitating hypothesis testing, predictions, and decisions. These models and their variations are heavily used in infectious disease management [13,14]. Simulations use these models to study processes or systems over time, enabling examination of different scenarios and their potential outcomes. Different types of simulations are immensely used in operations and resource management during major public health emergencies [15,16]. Visualization transforms data into graphical formats to communicate important messages [17,18]. Mapping and geographic information systems (GIS) analyze and present data based on geographic locations, usually using maps. GIS can be used to visualize and monitor infectious diseases, records, display populations’ healthcare needs, and manage available human and material resources [19]. The use of GIS-based mapping dashboards expanded during the COVID-19 pandemic [20,21]. Adaptation of these technologies in PHEOCs is very crucial for effective management of pandemics and health emergencies [22].

Recent studies on the use of digital technologies during the COVID-19 pandemic suggest the urgent need for studying and revisiting the weaknesses and strengths of these technologies across different contexts [23]. This study aims to identify specific scientific MSVM tools used by PHEOCs during the COVID-19 pandemic for decision-making and operations. It will examine recommended, best, and current practices in the application of MSVM during an infectious disease outbreak, assessing the benefits and limitations experienced by PHEOCs and how the use of MSVM tools evolved over the course of the COVID-19 pandemic. Although public health agencies have been using EOCs for managing health emergencies, including pandemics, the applications of MSVM have not been fully explored. In the absent of such studies, this study offers some valuable insights by conducting a systematic gap analysis based on survey data.

The rest of this paper is organized as follows: Section 2 presents the materials and methods used in the study. Section 3 presents the main results. Section 4 discusses the findings and explains some of the study’s limitations. Section 5 concludes the paper.

## 2. Materials and Methods

### 2.1. Questionnaire Design

We developed a comprehensive questionnaire for data collection, comprising 31 questions. Seven questions aimed to collect demographic information about the respondents, and the final two sought consent to share follow-ups of the research (Appendix A). The core of the survey included a mix of multiple-choice, Likert scale, and short-answer questions. The short-answer questions were added to identify demographic information or served as follow-up questions to multiple-choice questions, allowing respondents to contribute contextual information or provide additional information. These responses were coded to identify recurring themes. The survey concluded with two short-answer questions at the end, one allowing for any additional thoughts and the other for feedback on the survey itself. This survey was hosted on the Survey123 platform from ESRI to allow for information security and to use the advanced analytical capabilities of the platform.

### 2.2. Pilot Survey in Ontario

A revised draft of the survey was then piloted with four public health agencies in Ontario, Canada, to evaluate the clarity and relevance of the questions for current public health emergency management professionals. The feedback obtained was then incorporated into the final version of the survey, which was distributed to public health agencies across Ontario through the Ontario Public Health Emergency Management Network.

### 2.3. National Survey in Canada

The survey was also sent to public health agencies in all provinces of Canada using publicly available contact information. Overall, we received responses from three provinces (Ontario, Manitoba, and Alberta) and the Northwest Territories. Participants were initially given a total of 20 days to respond to the survey. Considering the busy schedule of the respondents during the ongoing COVID-19 pandemic, we extended the response period and made the survey available till January 2023. Respondents were provided with the option of responding through the link to the survey hosted on Survey123 or by completing a copy of the questionnaire provided as a Word document. Therefore, data collection was conducted between April 2022 to January 2023.

A total of 13 responses were received, 11 through the online survey platform and two from the Word document file. These responses were consolidated into a single dataset, which was then cleaned, coded, and analyzed. Employing basic frequency and descriptive statistical methods, we were able to achieve the study goals and objectives. The use of SMVM in PHEOCs and the identified gaps are presented and discussed in the remaining parts of the paper.

## 3. Results

### 3.1. Demographics

For this study, public health units in all provinces across Canada were contacted for the following study. The provinces of Ontario, Manitoba, Alberta, and the Northwest Territories were the primary participants in the study. We received 13 completed responses from the EOCs of the public health units (PHUs). Specifically, 34 units in Ontario were contacted, yielding 10 responses, which represents about 29% of the total public health units. The other three samples cover responses from Manitoba, Alberta, and the Northwest Territories (Table 1).

Among the survey participants, 76% reported working exclusively within their designated EOC, while the remaining attended multiple EOCs or had alternative arrangements for the COVID-19 pandemic response. Responses also included cases where respondents attended both an internal PHUEOC and upper Tier PHEOC or another case where the agency operated within their own agency and an EOC led by another agency. Regarding the mode of operation, about 78% indicated that the COVID-19 response operations were conducted either entirely or predominantly through remote means. More respondents reported two or more years of experience in their current role. The size of the PHEOC teams ranged from as few as nine to as many as 150. About 23% of respondents indicated that their EOC team had ten or fewer staff members, and one reported an EOC team larger than 100 members. Only two out of 13 reported that their jurisdiction served a population size of greater than 1,000,000 people.

The rapid spread and uncertainties surrounding the COVID-19 pandemic compelled many to shift to remote work to minimize their staff exposure to the virus. This transition was evident for public health agencies and units, with a significant portion of the respondents indicating that their operations shifted to being predominantly or entirely remote. We are not aware of how many of these agencies were using remote operations before the onset of the pandemic. Further research can shed light on the preparedness level of public health agencies for such major shifts.

### 3.2. Data Source for MSVM Analysis

Survey respondents were asked about the origin of the data used in their PHEOC’s MSVM analysis. The most common sources mentioned were “other government agencies” and “data collected or developed by public health agencies”, followed closely by “data collected by local medical facilities”. The next most notable data source was private organizations (72%). Only three organizations reported gathering data from non-profit organizations. The respondent who selected the category “others” specified that they used wastewater surveillance program data. It was noted that all participants utilized at least three data sources for their MSVM analysis (Table 2).

Considering that multiple respondents previously indicated using MSVM tools to analyze wastewater data, further inquiry into why the respondents felt that wastewater surveillance did not fit in one of the provided categories could provide beneficial context. Participants were able to select multiple data sources, so the number of respondents presented does not total eleven.

### 3.3. MSVM Application

**Figure 1:**
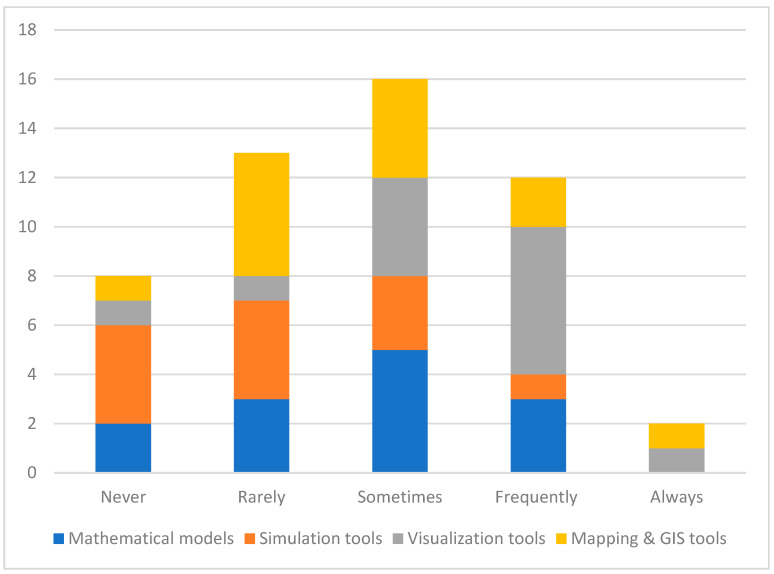
Frequency of overall MSVM use (Never: tools are not used at all; Rarely: tools are used infrequently; Sometimes: tools are used regularly but not consistently; Frequently: tools are used regularly but not all the time; Always: tools are used all the time).

Survey participants were queried on the frequency of SMVM tools in their PHEOCs during the COVID-19 pandemic. The findings presented in Figure 1 display a diverse level of usage. As indicated, most EOCs “sometimes” use the MSVM tools. Notably, visualization tools emerged as more frequently used tools. Simulation tools are among the tools that are never or rarely utilized. A segment of the respondents, six in total, reported frequent use of mathematical models. Most respondents selected “never” or “sometimes” when asked how frequently simulation tools were used in their EOC, with four answers each. Regarding GIS and mapping tools, moderate usage was reported with five “always” responses. Finally, five out of 12 public health EOC units reported “sometimes” for the use of mapping and GIS tools.

**Figure 2:**
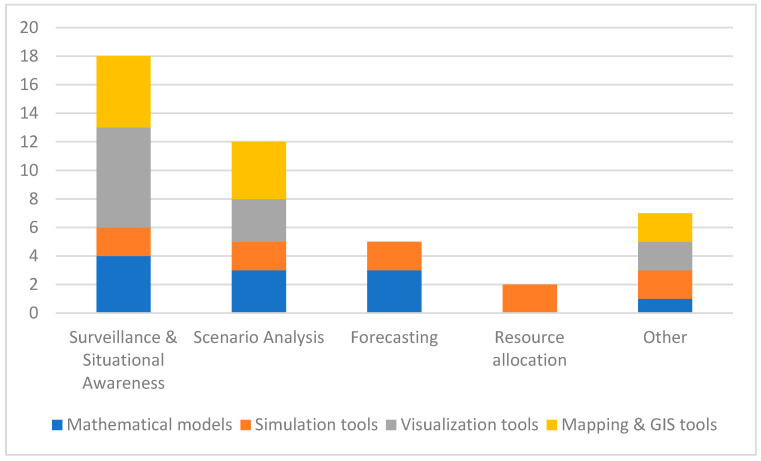
Use of MSVM tools in EOCs by purpose of use.

Figure 2 illustrates the results for the main purpose of using different MSVM tools within PHEOCs. Findings reveal that “Surveillance & Situational Awareness” is one of the key domains for MSVM tool application, followed by “Scenario Analysis”.

**Figure 3:**
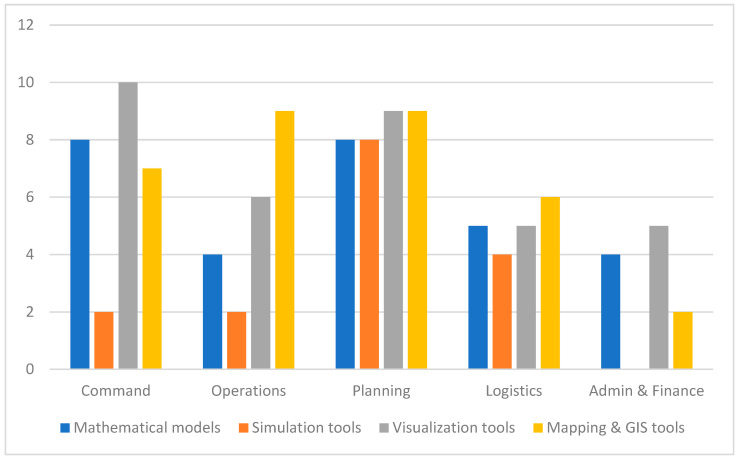
Use of MSVM tools in EOCs by EOC sections.

The use of MSVM tools in PHEOCs during the COVID-19 pandemic was further contextualized by asking respondents to identify which tools were mostly used by each of the PHEOC sections. The findings are presented in Figure 3. The findings reveal predominant use of visualization tools by the Command and Planning sections. Notably, Mapping and GIS tools were more favorable by the Operations sections. Consistent with the previous observations, simulation tools were less used across the board except for the Planning section. Visualization tools appeared to be the most frequently used MSVM tool across Canada in all PHEOC sections, followed by mathematical modeling and mapping and GIS tools. Simulation tools were significantly less utilized in PHEOC operations.

**Figure 4:**
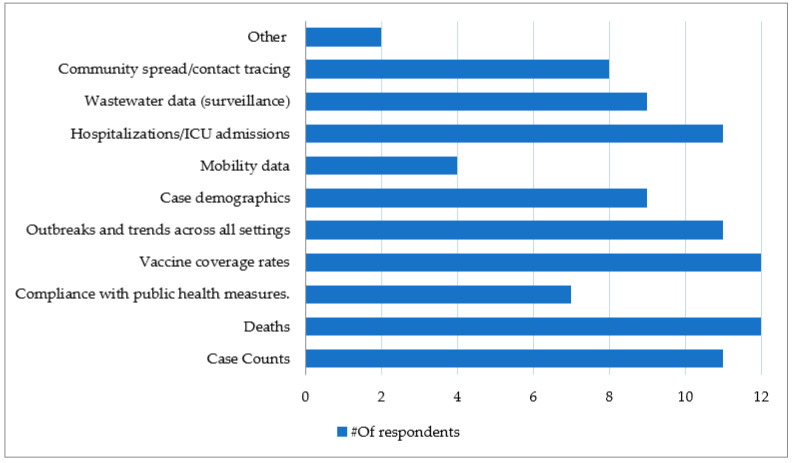
Types of information analyzed in EOCs using MSVM tools (number of responses = 13).

Figure 4 illustrates the types of information analyzed in the PHEOCs using MSVM tools during the response to the COVID-19 pandemic. The data most frequently subjected to analyses by the MSVM tools were Deaths and Vaccine coverage across all provinces. These were closely followed by Case counts, Outbreaks, and Trends across all settings and Hospitalizations/ICU admissions (91%). Finally, Case demographics, Wastewater data (surveillance) and Community spread/contact tracing were mentioned as the data used for analysis by the MSVM tools. “Other” responses noted in the questions included Compliance with public health measures and Mobility data. Some of the respondents chose “other” and further specified the types of information that were analyzed, such as exposure data to identify potential clusters and high-risk settings including abattoirs, student housing, and places of worship.

**Figure 5:**
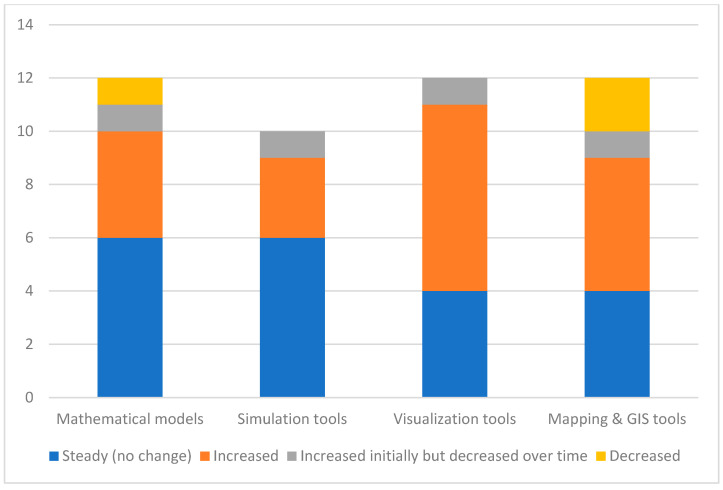
Reported change in the use of MSVM tools in EOCs.

Respondents were asked about the evolution in the use of MSVM tools in their PHEOC during the pandemic. These responses are displayed in Figure 5. A majority of the respondents noted that the use of Mathematical models remained steady with no change, and one reported an increase followed by a decrease in the use of Mathematical models. The use of simulation tools displayed similar pattern in that there was no change for most PHEOCs. One respondent noted an initial increase and then a decrease in the use of simulation tools. Four agencies did not answer the question for simulation tools, and two did not answer for Mathematical modeling, resulting in missing data.

Regarding Visualization tools, four respondents noted a steady use in PHEOC operations. One agency did not respond to the question. The findings for Mapping and GIS tools noted only one PHEOC reporting an “increase”. Others reported “steady (no change)” or an initial increase and then a decrease in the use of the tools. 

Further research could delve deeper into the specific use of MSVM tools in each PHEOC section, exploring the key tasks or activities for which these tools are employed. This exploration could reveal or highlight areas where MSVM tools are underutilized. The observed increase in the use of MSVM tools among the majority of respondents suggests a growing need for data analysis to support decision-making as the trajectory of the pandemic unfolded, or it could also indicate capacity building throughout the pandemic. The number of responses in favor of capacity building when addressing barriers could indicate a desire to prepare PHEOCs further, prior to future emergency responses. Few PHEOCs replied “very confident” when asked to rate their ability to apply MSVM tools. Future research on this topic might benefit from a more granular approach, asking respondents to rate their PHEOC staff’s confidence in developing, applying, and understanding/interpreting each separate MSVM tool, to understand further exactly where the gaps in technical ability might exist.

Three successive questions in the survey asked respondents to rate their PHEOC staff’s confidence in developing, applying, and understanding/interpreting MSVM analysis in their work. While responses showed some variations in the ratings, most respondents consistently reported that their PHEOC staff were “very confident” in their abilities regarding the MSVM tools. All responses indicated that their PHEOC staff were “somewhat” confident in developing MSVM tools. Most participants reported feeling “very confident” in using the MSVM analysis. Only two reported “less confident”. However, in terms of applying and understanding/interpreting the MSVM analysis, agencies reported a variety of confidence levels, including “less confident”, “somewhat confident”, and “very confident”. This suggests a notable discrepancy in confidence levels, with staff feeling more confident in developing MSVM analysis than in applying and understanding/interpreting the analysis.

### 3.4. Gap Analysis

**Figure 6:**
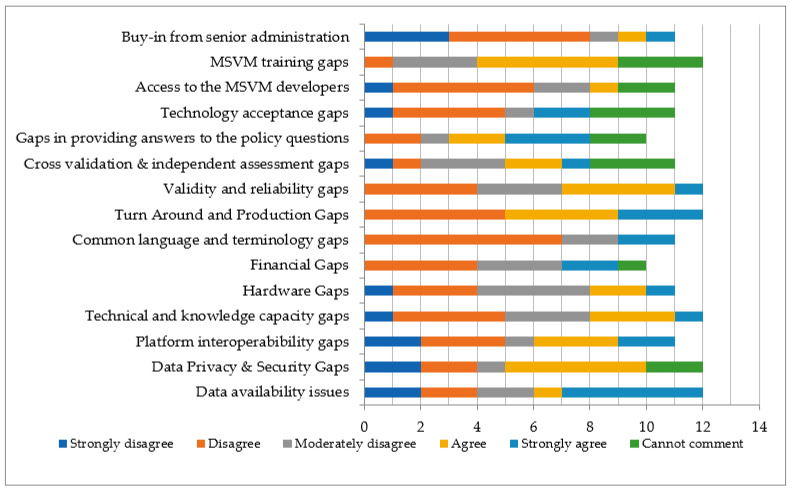
Gaps and barriers in use of MSVM in PHEOCs.

In understanding the challenges faced by the respondents in using MSVM tools during the COVID-19 pandemic, the survey asked for feedback on a range of barriers identified in the literature review. The results presented in Figure 6 reveal consensus around several gaps and barriers. These gaps can be divided into three categories: data gaps, technical gaps, and policy gaps.

Gaps in Data Privacy & Security as well as issues obtaining buy-in from senior administration were prevalent. These two categories had the highest number of “agree” and “strongly agree” responses. More than half of the respondents also agreed to some degree that there were validity and reliability gaps present in the use of MSVM tools in their EOC during the COVID-19 response. Participants noted both a lack of local data, as well as too much available information, as barriers. There was also mention of inconsistent data formats creating challenges, indicating issues with interoperability.

Most of the respondents disagreed to some extent about having any technical or knowledge capacity gaps. Four agencies strongly agreed or agreed that they experienced these gaps. Most respondents noted gaps in turnaround, production, and platform interoperability. Many respondents also agreed to some extent that their EOC faced MSVM training gaps during their COVID-19 response. One respondent expressed a need for access to application programming interface (API) connections to all public health organizations to improve opportunities for automation and efficiency. This can also be linked to data integration and the creation of a common platform among the PHEOCs [12]. Finally, it was mentioned that mapping was challenging in rural areas because of the wider population that did not always fit within municipal boundaries.

Half of all respondents also agreed to some extent that there were gaps in providing answers to policy questions during their COVID-19 response. Most respondents noted “disagree” and “moderately disagree” for the category of financial gaps or constraints. Participants were also allowed to identify additional barriers they had encountered in a follow-up question. Analysis of the responses revealed the presence of both data issues and technology issues when using MSVM tools in respondents’ COVID-19 responses.

## 4. Discussion

Our findings suggest the existence of technological gaps in PHEOCs, which vary between different EOCs. The elevated level of support for the proposed interventions suggests keen motivation among all respondents and the broader public health emergency management community in Canada to improve the efficacy and accessibility of MSVM tools in PHEOCs.

The strong support for creating standard tools indicates a need for increased interoperability among agencies, which could facilitate knowledge and experience sharing and allow decision-makers in different jurisdictions to create cohesive and complementary policies. This is an area that perhaps the Public Health Agency of Canada or provincial public health agencies can take a lead on that and facilitate the creation of such standard tools.

Despite all respondents disagreeing to some extent that financial gaps or constraints existed in the use of MSVM in the COVID-19 response, the creation or award of grants to local-level EOCs was a popular way to address gaps and barriers. This might indicate that financial capacities have been adequate but could be improved. Strengthening EOCs’ capacities to leverage digital technologies based on the lessons learned from the COVID-19 pandemic, and making these technologies more readily available for robust decision-making can contribute to better health outcomes [23].

Several participants suggested that addressing collaboration and interoperability gaps could be achieved by building upon existing networks. This approach, which prioritizes building on the current networks over creating new ones, presents a more resource-efficient way to improve collaboration as well as interoperability across PHEOCs. Further research into this topic could delve into the existing networks to assess their effectiveness, scope, formality levels, and potential interests in collaboration and expansion. Such investigation would be very valuable for identifying opportunities for partners who have not been involved in such networks or would like to be more directly involved.

While language and terminology barriers were specifically mentioned by only two respondents, the additional feedback sections highlighted that resources to overcome these challenges would be beneficial for effective communication with the end-users, decision-makers, and the public. Although this concern seems minor relative to other challenges, two out of seven responses is not insignificant. Therefore, this area warrants further investigation to identify and develop solutions that can address these barriers to enhance understanding and engagement of all stakeholders and units involved in and working with the PHEOCs.

Respondents were dealing with emergency response operations for an ongoing global pandemic at the time the survey was conducted. In the interest of not alienating participants who might otherwise be too busy to participate, the survey was made as short and concise as possible, which often included asking questions relating to MSVM tools as a broad group rather than asking individual questions for each type of MSVM tool. This lack of detail limits the conclusions that can be drawn from the results of this survey, since it does not allow us to capture the full context of each response. Future research could add some follow-up open-ended questions or conduct semi-structured interviews to capture more details.

It is important to note that because this sample is not large, it is likely that these results may not encapsulate all diverse PHEOCs in Canada. Rather, they provide some insights and serve as a first step of research in examining the technological needs of PHEOCs based on the experiences of COVID-19, a pandemic that activated all of them.

The survey’s insights are subject to some limitations that warrant consideration. Primarily, the small sample size (even though it is relatively good compared to the total number of PHEOCs in Canada), may not be representative of all EOCs. We suspect that this relatively low response was caused by the fact that many potential respondents were dealing with emergency response operations for the ongoing global pandemic when the survey was conducted. Also, while we tried to make the survey as concise as possible, the survey still included broad questions relating to MSVM tools rather than specific questions for each MSVM tool. Nevertheless, this survey reached the designed goal of presenting the problems of digital applications during the pandemic, and this is a valuable chance to push forward and utilize digital transformation in emergency management.

## 5. Conclusions

Public Health Emergency Operations Centers (PHEOCs) are focal points for managing health emergencies, including pandemics. Their effectiveness in part depends on access to and capacity for using digital tools. Understanding the current state of digital tool utilization by PHEOCs can provide key information for developers and PHEOC managers. This study performed a survey on the use of digital tools, classified into modeling, simulation, visualization, and mapping (MSVM), by PHEOCs in Canada. Findings of the study revealed that PHEOCs use Visualization and Mapping tools more than Modeling and Simulation tools. These tools are primarily used for surveillance, situational awareness, and scenario analysis. Among the PHEOC sections, digital tools are mostly used by the Command, Planning, and Operations sections. While the usage of different digital tools has fluctuated over the study period, our findings showed an increasing trend in the use of digital tools by PHEOCs. This reflects the increasing need for data analysis, as well as increasing capacities and confidence in using such tools.

However, critical gaps exist in some areas, including data management and integration, technical capacity, interoperability, and digital policymaking. The use of digital tools generates data privacy and security issues and requires buy-in from senior administration. There were also concerns about the validity and reliability of the output generated by digital tools during the COVID-19 response. Learning from how digital tools were used during the COVID-19 pandemic by PHEOCs and understanding their challenges, new generations of MSVM tools should be developed to further enhance PHEOCs’ toolsets. Similar to many other areas, public health and PHEOCs are undergoing digital transformation. By learning from the COVID-19 pandemic and leveraging advancements that have been made in digital technologies, PHEOCs can be equipped with the next generations of digital tools, making the management of public health emergencies more effective, data-driven, evidence-based, and transparent. More studies are required to redefine the next generations of PHEOCs and how digital transformation will shape them in a more standard synergic way.

## Figures and Tables

**Table 1:** Demographic information of survey respondents (source: authors’ survey data).

Item	Category	Response
Location	Operated within own EOC within the PHU	10
	Operated in multiple EOCs	1
	Other	2
Work pattern	Entirely Remote	4
	Mostly Remote	6
	Mix (half/half)	3
Experience in current role	Up to 2 years	5
	3–5 years	3
	5+ years	5
Number of employees in EOC	Up to 10	3
	11–100	8
	>100	1
Size of Jurisdiction	<1,000,000 people	11
	>1,000,000 people	2

**Table 2:** Data sources used for MSVM analysis (source: authors’ survey data).

Organization	Number
Government agencies	11
Data collected or developed by public health	11
Local medical facilities	10
Private organization	8
Non-profit organization	3
Other	3

## Data Availability

Unaggregated data for this study are not available as per our research ethics certificate.

## Appendix A. Questionnaire

Using MSVM in HEOCs during the COVID-19 pandemic
Date: 22 March 2022
Study Name: Understanding Modelling, Simulation, Visualization, and Mapping Gaps in Public Health Emergency Operations Centers during the COVID-19 Pandemic

Definitions:
Mathematical modeling: Mathematical models are a representation of objects and their behaviors in mathematical terms, used to make hypotheses, predictions, or decisions. Ex. Use of forecasting models to predict case numbers.
Simulation software: Simulation is the use of models to observe a process or system over time, allowing users to explore different scenarios and outcomes. Ex. Using a simulation to evaluate the best way to allocate resources.
Visualization: Visualization is the practice of presenting data in a graphic format to communicate a message. Ex. A publication describing trends in case numbers.
Mapping and GIS: Mapping and geographic information systems present data based on geographic locations, usually using maps. Ex. A dashboard that tracks vaccine uptake in various geographical regions.

1. Which Public Health Agency (PHA) or Public Health Unit or Region (PHU/PHR) are you responding on behalf of? Please note that this is for internal use, and we will not publish the results of the study in an identifiable manner.
2. Within what type of Emergency Operations Center (EOC) did your PHA/PHU/PHR operate during the COVID-19 pandemic?
  We operated within our own agency’s Health Emergency Operations Center (HEOC).	
  We operated within an Emergency Operations Center (EOC) led by our jurisdiction (ex. Municipal Emergency Operations Centre).	
  We operated in an EOC led by someone else (not our agency or jurisdiction).	
  We operated in multiple EOCs.	
  We did not operate within an Emergency Operations Center (EOC).	
  Other (Please Specify)	
3. How did your HEOC/EOC operate during the COVID-19 pandemic?
  All operations were conducted remotely	
  Most operations were conducted remotely	
  About half of operations were conducted remotely	
  Few operations were conducted remotely	
  The HEOC/EOC did not conduct any operations remotely	
  Other (Please Specify)	
4. What is/was your role in the HEOC/EOC?
5. How long have you been/were you in this role?
6. On average how many people work or worked in your HEOC/EOC?
7. What is the size of the population your HEOC/EOC serves?
8. How often did you use the following MSVM tools in your HEOC/EOC during the COVID-19 response?
  	Never	Rarely	Sometimes	Frequently	Always
  Mathematical models					
  Simulation tools					
  Visualization tools					
  Mapping and GIS tools					
9. Which functions/activities were the following MSVM tools used for in your HEOC/EOC during the COVID-19 response?
  	**Forecasting**	**Support Data Driven Decision Making and Scenario Analysis**	**Surveillance**	**Resource Allocation**	**Situational Awareness**	**Inform Communications Strategy**	**Not Applicable**
  Mathematical models							
  Simulation tools							
  Visualization tools							
  Mapping and GIS tools							
10. What information was analyzed using MSVM in your HEOC/EOC during the COVID-19 response? Please select all that apply.
  Case counts	
  Deaths	
  Compliance with public health measures	
  Vaccine coverage rates	
  Outbreaks and trends across all settings (institutional, congregate, workplace, schools, etc.)	
  Mobility data	
  Case demographics	
  Hospitalization, ICU admissions	
  Wastewater data (surveillance)	
  Community spread/contact tracing	
  Other (please specify)	
11. Which of the following MSVM tools were mostly used by the HEOC/EOC Command Section? You can choose more than one option.
  Mathematical models	
  Simulation tools	
  Visualization tools	
  Mapping and GIS tools	
  None of the above	
12. Which of the following MSVM tools were mostly used by the HEOC/EOC Operations Section? You can choose more than one option.
  Mathematical models	
  Simulation tools	
  Visualization tools	
  Mapping and GIS tools	
  None of the above	
13. Which of the following MSVM tools were mostly used by the HEOC/EOC Planning Section? You can choose more than one option.
  Mathematical models	
  Simulation tools	
  Visualization tools	
  Mapping and GIS tools	
  None of the above	
14. Which of the following MSVM tools were mostly used by the HEOC/EOC Logistics Section? You can choose more than one option.
  Mathematical models	
  Simulation tools	
  Visualization tools	
  Mapping and GIS tools	
  None of the above	
15. Which of the following MSVM tools were mostly used by the HEOC/EOC Admin and Finance Sections? You can choose more than one option.
  Mathematical models	
  Simulation tools	
  Visualization tools	
  Mapping and GIS tools	
  None of the above	
16. Overall, what factors influenced the decision to use the MSVM tools that your HEOC/EOC currently uses? You can choose more than one option.
  Cost	
  Interoperability with partners/stakeholders	
  Familiarity of staff with the tools	
  Recommendation	
  Functionality	
  Ease of use	
  Reliability	
  Other (Please Specify)?	
17. How did the use of MSVM tools change in your HEOC/EOC during the COVID-19 pandemic (From February 2020 to February 2022)?
  	**Increased over Time**	**Steady (no Change) Throughout**	**Decreased**	**Increased Initially, but Decreased Over Time**
  Mathematical models				
  Simulation tools				
  Visualization tools				
  Mapping and GIS tools				
18. In your opinion, how effectively were each of the following functions/activities conducted in your HEOC/EOC?
  	**Not effective at All**	**Slightly Effective**	**Moderately Effective**	**Very Effective**	**Extremely Effective**	**Not Applicable**
  Forecasting						
  Support data driven decision making and scenario analysis						
  Surveillance						
  Resource Allocation						
  Situational Awareness						
  Inform communications strategy						
19. From what sources was data collected for MSVM analysis in your HEOC/EOC during the COVID-19 response? Please select all that apply.
  Local medical facilities (e.g., hospitals)	
  Government agencies (e.g., Ministry of Health)	
  Data collected or developed by public health	
  Private organizations (e.g., private laboratories)	
  Non-profit organization (e.g., universities, Red Cross)	
  Other	
20. Were MSVM functions conducted by the HEOC/EOC employees or an external party in your HEOC/EOC during the COVID-19 response? Please select all that apply.
  	**EOC/HEOC Staff**	**Public Health Unit/Agency Staff**	**Other Staff in the Jurisdiction**	**External Providers/Universities**	**External Providers/Private Sector**	**Not Applicable**
  Mathematical models						
  Simulation tools						
  Visualization tools						
  Mapping and GIS tools						
21. Please state if you agree or disagree that the following gaps and barriers were present in the use of different MSVM tools in your HEOC/EOC.
  **Gaps & Barriers**	**Strongly Disagree**	**Disagree**	**Moderately Disagree**	**Agree**	**Strongly Agree**	**Cannot Comment**
  Data availability issues						
  Data privacy & security gaps						
  Platform interoperability gaps						
  Technical and knowledge capacity gaps						
  Hardware gaps						
  Financial gaps						
  Common language and terminology gaps						
  Turn around and production gaps						
  Validity and reliability gaps						
  Cross validation & independent assessment gaps						
  Gaps in providing answers to the policy questions						
  Technology acceptance gaps						
  Access to the MSVM developers						
  MSVM training gaps						
  Buy-in from senior administration						
22. Were there any other gaps or barriers present in the use of di
23. Overall, how satisfied were your HEOC/EOC members with existing MSVM solutions?
  	**Not at All Satisfied**	**A Little Bit Satisfied**	**Somewhat Satisfied**	**Very Satisfied**	**Completely Satisfied**
  Mathematical models					
  Simulation tools					
  Visualization tools					
  Mapping and GIS tools					
24. Rate the confidence of your HEOC/EOC staff or core program staff in developing the MSVM analysis that they use.
  Not at all confident	
  A little bit confident	
  Somewhat confident	
  Very confident	
  Completely confident	
25. Rate the confidence of your HEOC/EOC staff in applying MSVM analysis to their regular tasks in the COVID-19 response.
  Not at all confident	
  A little bit confident	
  Somewhat confident	
  Very confident	
  Completely confident	
26. Rate the confidence of your HEOC/EOC staff in understanding/interpreting the MVSM analysis that they used.
  Not at all confident	
  A little bit confident	
  Somewhat confident	
  Very confident	
  Completely confident	
27. Thinking about how your HEOC/EOC used MSVM during the COVID-19 response, do you agree that the following statements are effective ways that some of the existing gaps can be reduced or closed?
  	**Strongly Disagree**	**Disagree**	**Moderately Disagree**	**Agree**	**Strongly Agree**	**Cannot Comment**
  Creating standard national MSVM tools to be used by HEOCs/EOCs						
  Creating standard provincial MSVM tools to be used by HEOCs/EOCs						
  Creating a national support centre for MSVM tools for the HEOCs/EOCs						
  Creating a national university-based research centre to support the use and development of MSVM tools for Canadian HEOCs/EOCs						
  Creating national/regional knowledge/experience sharing platforms for use of MSVM tools in HEOC/EOC						
  Providing grants to regional/local HEOC/EOCs to enhance their MSVM tools						
  No additional support is needed						
28. Are there other ways that some of the existing gaps can be reduced or closed?
29. If you have any additional comments or suggestions about the above questions or this research that you would like to share, please use the following box to share it with us.
30. Would you like to receive the results of this study once completed?
  Yes	
  No	
31. If you answered “Yes” to the above question, please provide your email to receive the results.
